# Supplementary material for: Origin of slow earthquake statistics in low-friction soft granular shear
Source: Nat Commun. 2025 Dec 1;16:10236. doi: 10.1038/s41467-025-65230-z (PMC12669249; doi:10.1038/s41467-025-65230-z)
Supplement: Supplementary file 1 — Supplementary Information [file 41467_2025_65230_MOESM1_ESM.pdf]

Origin of slow earthquake statistics in low-friction soft granular shear

Yuto Sasaki[1*] and Hiroaki Katsuragi[1]

[1]Department of Earth and Space Science, The University of Osaka, 1-1, Machikaneyama, Toyonaka, 5600043, Osaka, Japan.

*Corresponding author(s). E-mail(s): sasaki.geoscience@gmail.com;

Supplementary information

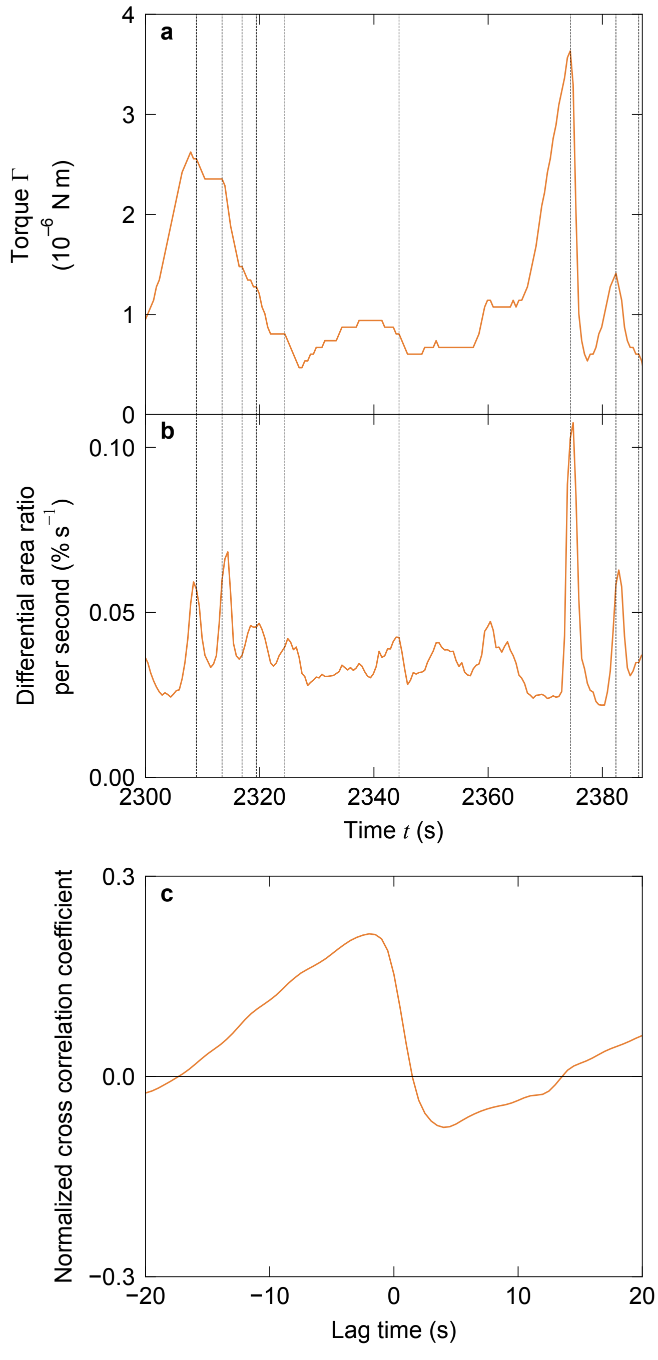

Supplementary Fig. 1: Correlation between the torque and particle motion. The data were obtained in run #83 ($\phi = 0.20$). (**a**) Torque data. The vertical dashed lines indicate the onset of each event defined by torque drop. (**b**) Differential area (particle motion area) per second, normalized to the whole area. The vertical dashed lines indicate the onset of each event defined by torque drop. (**c**) Normalized cross correlation coefficient between the torque and differential area ratio per second, calculated after subtracting the mean from each time series (t = 1890–2387 s). The correlation is plotted as a function of the lag time of torque relative to the differential area ratio (particle motion). The peak correlation at slightly negative lag time implies synchronization where the peak of accelerated particle motion follows the corresponding torque drop onset.

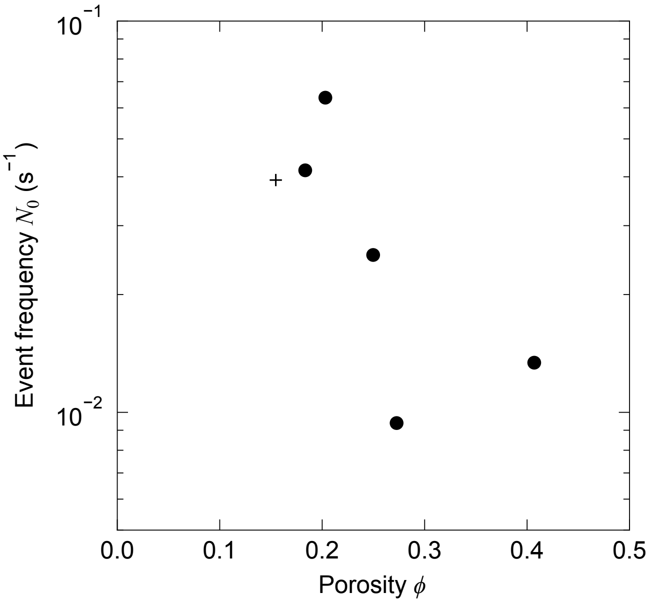

Supplementary Fig. 2: Event frequency as a function of porosity. Solid black circles represent the frequency of events (s^{-1}) obtained from exponential fitting (N_0 in Equation (3)). The cross symbol is a datum at the lowest porosity in run #73 ($0.15 \lesssim \phi \lesssim 0.18$), where many particles were not confined to the same monolayer. This exceptional datum is provisionally plotted at $\phi = 0.15$.

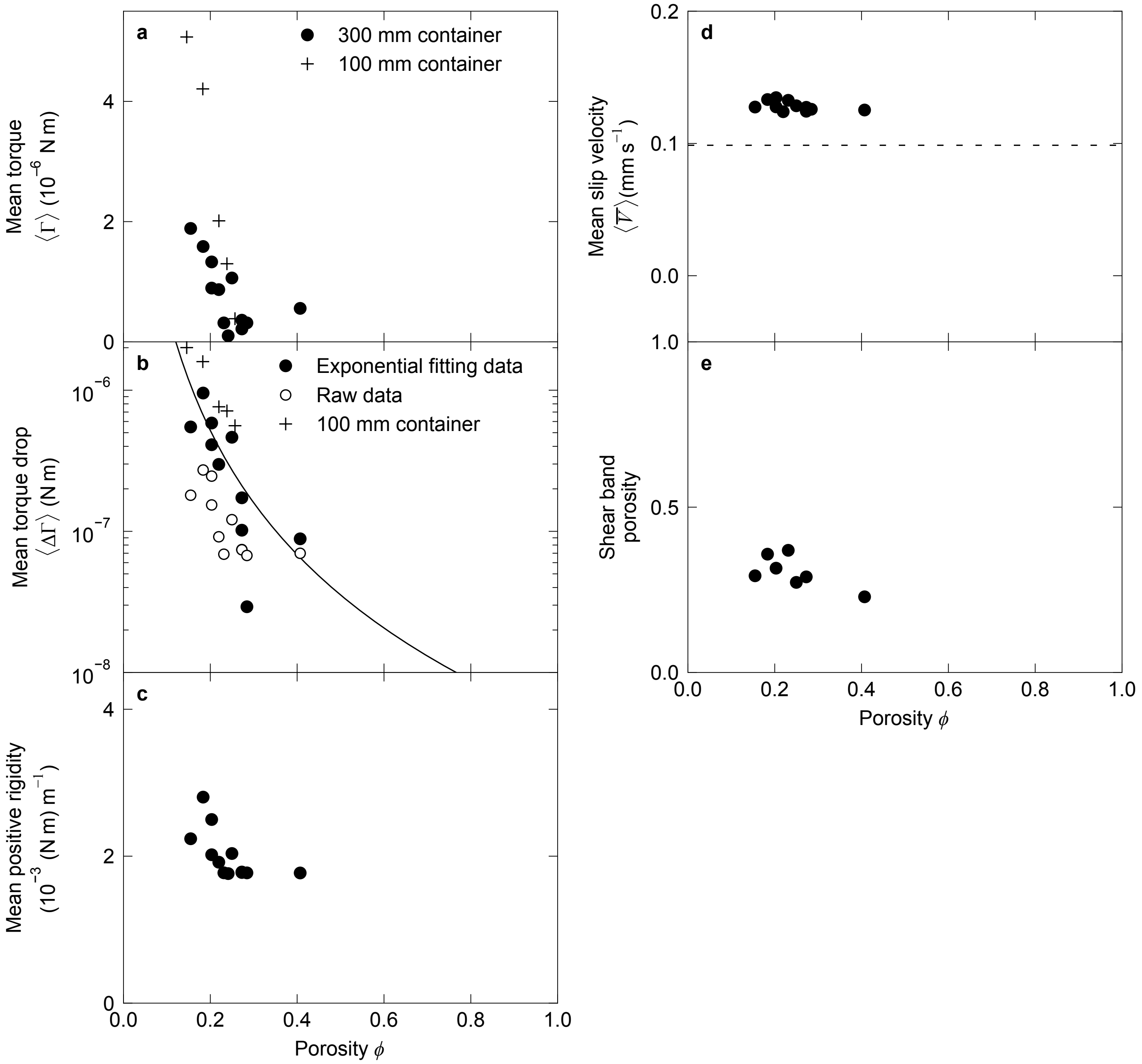

Supplementary Fig. 3: Porosity dependence of measured variables. (**a**) Mean torque. The black circles correspond to the mean data using the 300 mm container and the cross symbols are the data using the 100 mm container for comparison. (**b**) Mean torque drop amplitude. The solid circles correspond to the means obtained from the exponential fitting and the open circles are the means calculated from raw data in Supplementary Fig. 4. The cross symbols correspond to the means obtained from the exponential fitting using the 100 mm container. The solid fitting curve is represented by Equation (8) using $\Delta\sigma = A\phi^{-n}$ ($A = 4.7 \times 10^{-13}$ Pa, $n = 5.0$). (**c**) Mean positive rigidity calculated as the ratio of torque increment to displacement increment between adjacent time steps. (**d**) Mean tangential slip velocity at the cylinder surface. The dashed line indicates the constant motor velocity without any torque resistance. (**e**) Measured porosity within the shear band with a thickness w.

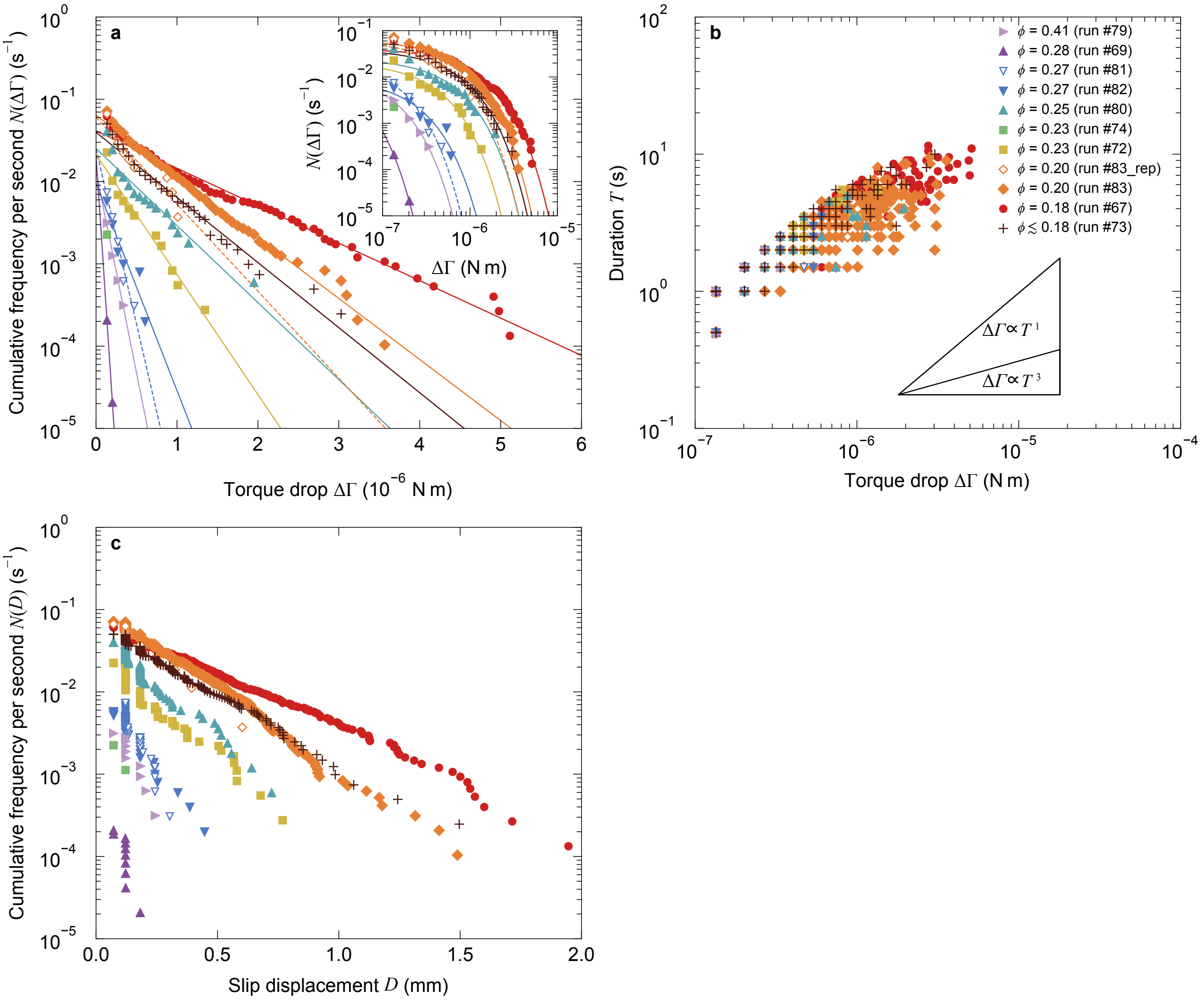

Supplementary Fig. 4: Statistical characteristics of torque drop events in the system with a diameter of 300 mm. The symbols and colors are common in all subplots. (**a**) Cumulative frequency distribution of the torque drop amplitude $\Delta\Gamma$. The inset shows the same data in a log-log plot. (**b**) Relationship between torque drop amplitude and duration. (**c**) Cumulative frequency distribution of the tangential slip displacement D at the cylinder surface.

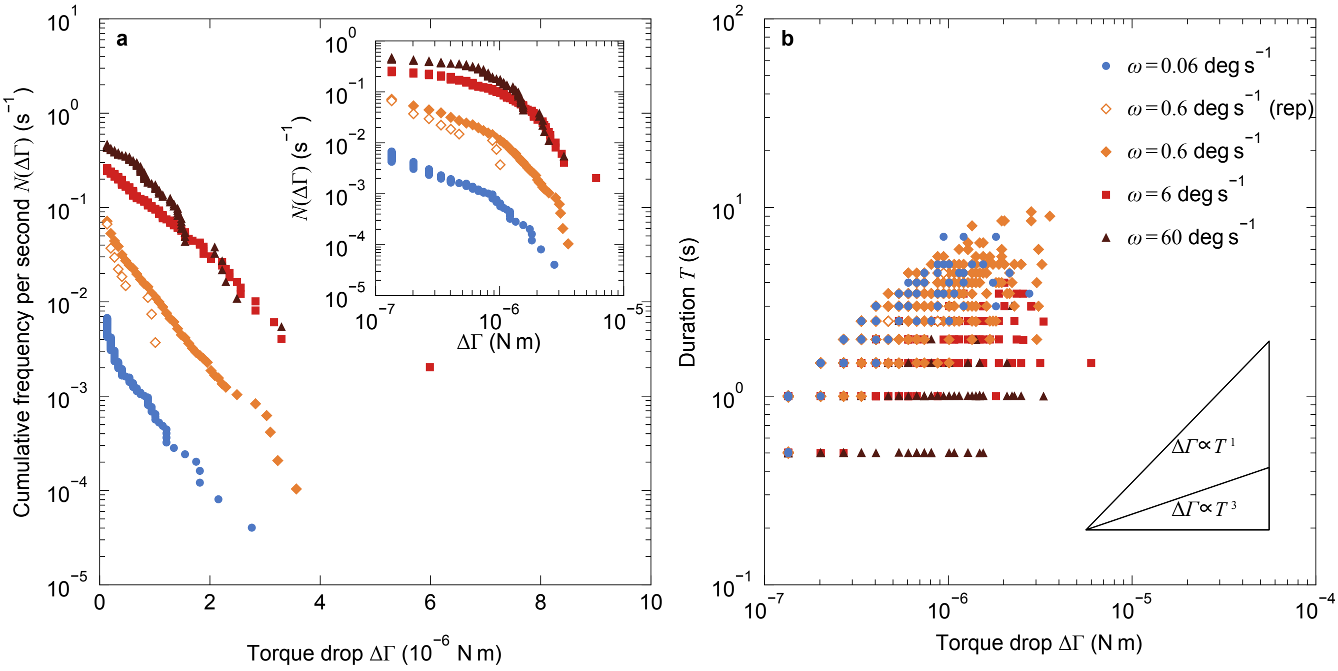

Supplementary Fig. 5: Statistical characteristics of torque drop events at various rotation rates. All data are obtained in run #83 ($\phi = 0.20$). (**a**) Cumulative frequency distribution of the torque drop amplitude $\Delta\Gamma$. The inset shows the same data in a log-log plot. The symbols and colors are the same as (b). (**b**) Relationship between torque drop amplitude and duration. The symbols and colors correspond to the rotation rates of $\omega = 0.06$–$60\ \mathrm{deg\,s^{-1}}$. The open symbols correspond to the replication test.

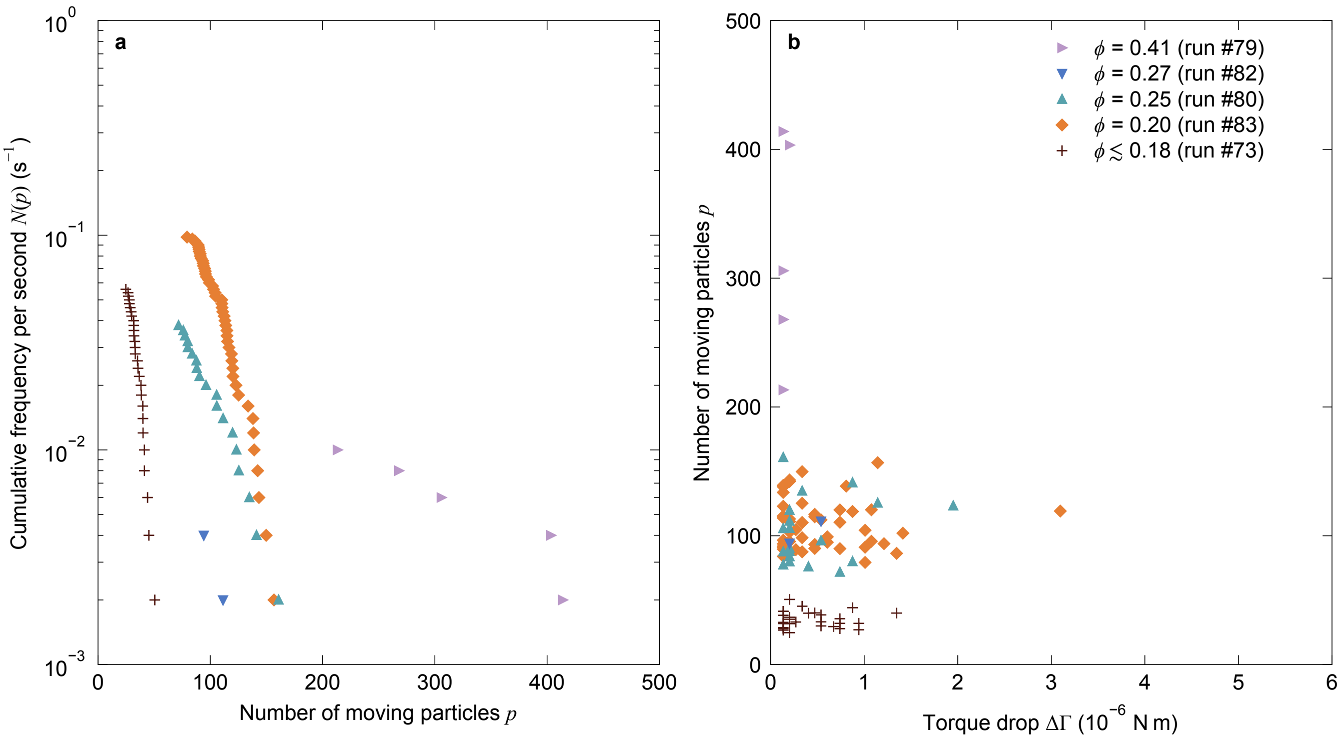

Supplementary Fig. 6: Distribution of the particle number corresponding to each slip events. The data were obtained from a 500-second segment taken more than 1000 s after the beginning of each run. (**a**) Cumulative frequency distribution of the number of moving particles p. The symbols and colors are the same as (b). (**b**) Number of moving particles p as a function of the torque drop amplitude $\Delta\Gamma$.

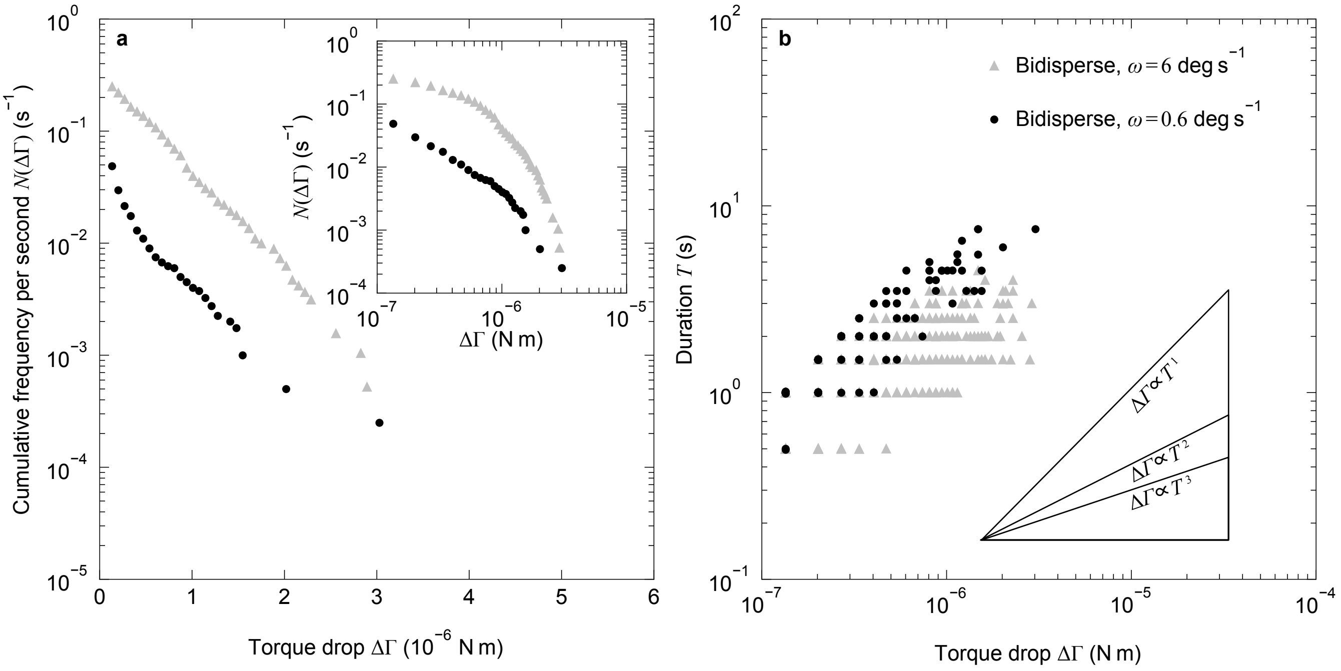

Supplementary Fig. 7: Statistical characteristics of torque drop events of bidispersed mixture of hydrogel particles and glass beads in the system with a diameter of 300 mm. The data were obtained from run #77 with d = 4.4 mm hydrogel particles and 2 mm glass beads, sheared at 0.60 and 6 deg s^{-1}. All data are shown in black and white due to out-of-plane particles and undefined porosity ($\phi < 0.18$), unlike other figures. (**a**) Cumulative frequency distribution of the torque drop amplitude $\Delta\Gamma$. The inset shows the same data in a log-log plot. The symbols and colors are the same as (b). (**b**) Relationship between torque drop amplitude and duration.

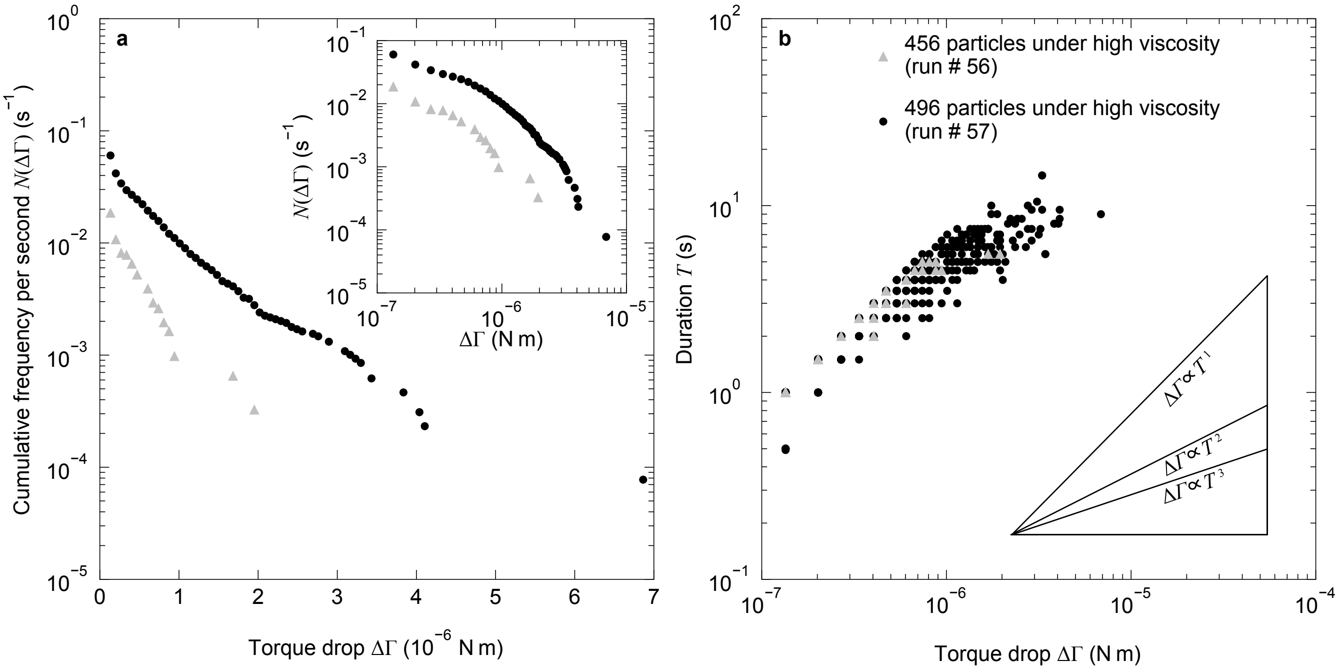

Supplementary Fig. 8: Statistical characteristics of torque drop events using viscous liquid and hydrogel particles in the system with a diameter of 300 mm. The data were obtained using the lubricating liquid with higher viscosity (1 Pa s). All data are shown in black and white due to out-of-plane particles and undefined porosity ($\phi < 0.18$, runs #56, 57), unlike other figures. (**a**) Cumulative frequency distribution of the torque drop amplitude $\Delta\Gamma$. The inset shows the same data in a log-log plot. The symbols and colors are the same as (b). (**b**) Relationship between torque drop amplitude and duration.

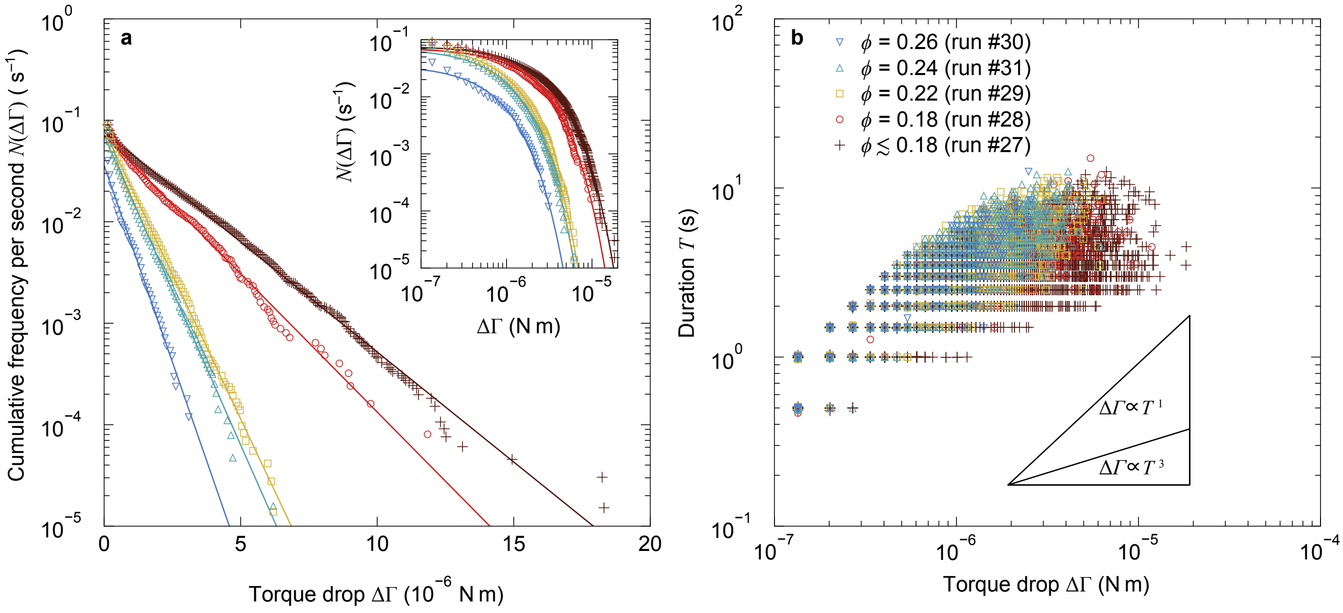

Supplementary Fig. 9: Statistical characteristics of torque drop events in the system with a diameter of 100 mm. The symbols and colors are common in all subplots. (**a**) Cumulative distribution of the torque drop amplitude $\Delta\Gamma$. The inset shows the same data in a log-log plot. The symbols and colors are the same as (b). (**b**) Relationship between torque drop amplitude and duration.

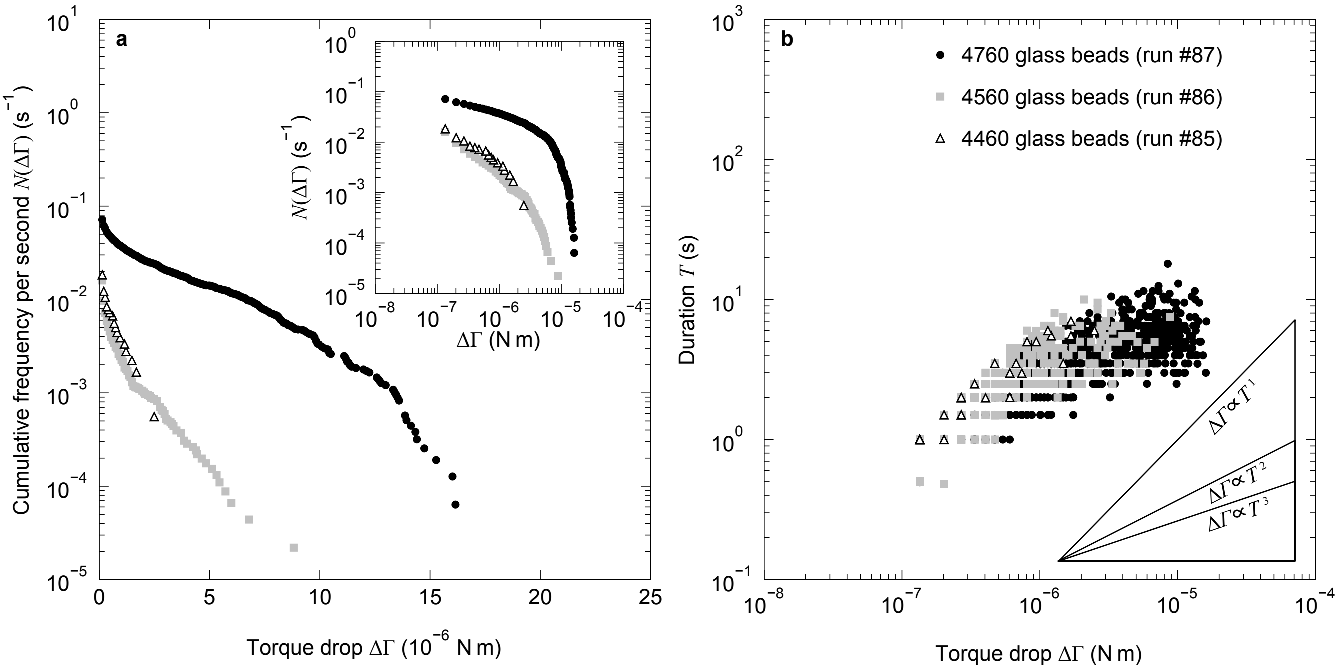

Supplementary Fig. 10: Statistical characteristics of torque drop events in the system with a diameter of 300 mm using glass beads. The data were obtained using 4460–4760 glass beads with a diameter of 4.1 mm. All data are shown in black and white due to out-of-plane particles and undefined porosity ($\phi < 0.18$, runs #85–87), unlike other figures. (**a**) Cumulative frequency distribution of the torque drop amplitude $\Delta\Gamma$. The inset shows the same data in a log-log plot. The symbols and colors are the same as (b). (**b**) Relationship between torque drop amplitude and duration.

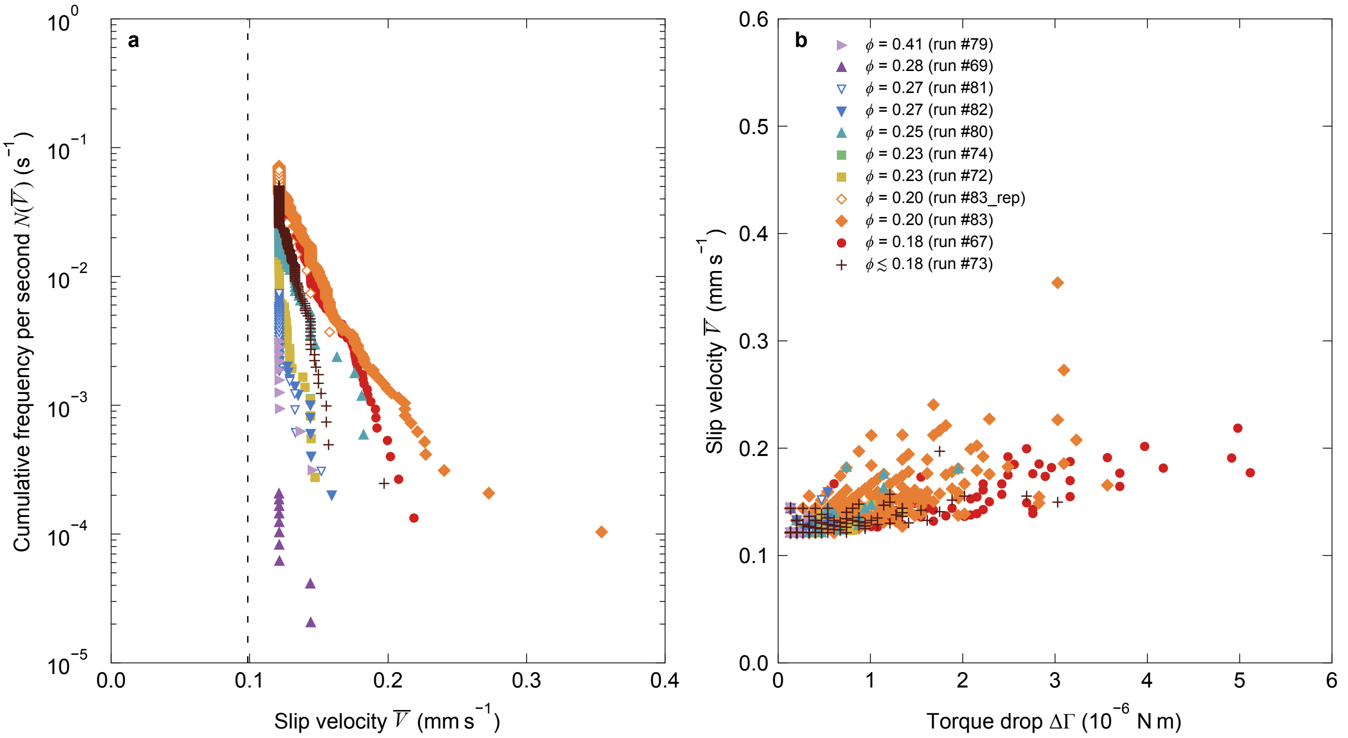

Supplementary Fig. 11: Slip velocity distribution as functions of porosity ϕ and torque drop amplitude $\Delta\Gamma$. The symbols and colors are common in all subplots. (**a**) Cumulative frequency distribution of the tangential slip velocity $\overline{V}$ at the cylinder surface. The dashed line indicates the constant motor velocity without any torque resistance, corresponding to the dashed line in Fig. 1d inset). The resolution of the calculated velocity is approximately 0.02 mm s^{-1} in this experimental system. Symbols and colors are the same as (b). (**b**) Torque drop dependence of the slip velocity.

Supplementary Table 1: Experimental conditions used for analysis at 0.60 $\deg\,s^{-1}$.

Run#	System size (mm)	Liquid viscosity (Pa s)	Number of particles[1]	Porosity	Run duration (s)	Detected events
79	300	0.02	2746 hydrogels	0.41	3194	10
69	300	0.02	3314 hydrogels	0.28	48704	10
81	300	0.02	3368 hydrogels	0.27	3264	24
82	300	0.02	3368 hydrogels	0.27	4984	29
80	300	0.02	3474 hydrogels	0.25	1699	68
70[2]	300	0.02	3514 hydrogels	0.24	184	0
74	300	0.02	3560 hydrogels	0.23	885	2
72	300	0.02	3614 hydrogels	0.22	3628	82
83	300	0.02	3690 hydrogels	0.20	9630	690
83rep[3]	300	0.02	3690 hydrogels	0.20	267	18
67[2]	300	0.02	3781 hydrogels	0.18	7512	466
73[4]	300	0.02	3914 hydrogels	$\lesssim 0.18$	4052	204
30	100	0.02	401 hydrogels	0.26	16819	672
31[5]	100	0.02	411 hydrogels	0.24	63459	5103
29	100	0.02	421 hydrogels	0.22	72289	6493
28	100	0.02	441 hydrogels	0.18	12436	1130
27[4]	100	0.02	461 hydrogels	$\lesssim 0.18$	65821	5941
56[4,5]	100	1	456 hydrogels	$\lesssim 0.18$	3075	57
57[4,5]	100	1	496 hydrogels	$\lesssim 0.18$	12917	779
85[2,4]	300	0.02	4460 glass beads	N/A	9244	33
86[2,4]	300	0.02	4560 glass beads	N/A	45486	728
87[2,4]	300	0.02	4760 glass beads	N/A	15726	1125
77[4,6]	300	0.02	ca 6800 bidispersed	N/A	4010	195

[1]Number of particles including the particles glued to the rotating cylinder.

[2]Particles arranged in an ordered polycrystal structure.

[3]Performed at the end of run #83 to verify reproducibility.

[4]Several particles are positioned out of the plane of the granular layer.

[5]Including the particles without the immersion pretreatment.

[6]Using a bidispersed mixture of hydrogel particles and glass beads.
