# Supplementary material for: Origin of slow earthquake statistics in low-friction soft granular shear
Source: Nat Commun. 2025 Dec 1;16:10236. doi: 10.1038/s41467-025-65230-z (PMC12669249; doi:10.1038/s41467-025-65230-z)
Supplement: Supplementary file 2 — Description of Additional Supplementary File [file 41467_2025_65230_MOESM2_ESM.pdf]

Description of Additional Supplementary Files

Supplementary Movie 1:

Representative timeseries of particle motion. An animated version is displayed for the binary image differences with a 2-second interval in run #83, including the images in Fig. 3b, d, e. The white color shows the differences between the binary images with an interval of 2 s, indicating the areas swept by particles. The frame rate is 10 fps and the playback speed is the same as the actual speed. The playback times of 0, 337.7, 352.8, and 495.4 s correspond to the run times of 1890.1, 2229.3, 2244.5, and 2387.8 s (the difference in brightness values was calculated between two images taken ±1 s apart), respectively. Colors have been inverted for better visibility. Scale bar: 50 mm
